# MEFV gene mutations in Egyptian children with Henoch-Schonlein purpura

**DOI:** 10.1186/1546-0096-12-41

**Published:** 2014-09-09

**Authors:** Samia Salah, Samia Rizk, Hala M Lotfy, Salma EL Houchi, Huda Marzouk, Yomna Farag

**Affiliations:** Department of Pediatrics, Faculty of Medicine, Cairo University, Cairo, Egypt; Department of Clinical Pathology, Faculty of Medicine, Cairo University, Cairo, Egypt

**Keywords:** Familial Mediterranean fever, Henoch-Schonlein purpura (HSP), *MEFV*, Mutations

## Abstract

**Background:**

Due to an increased frequency of vasculitis in FMF patients, many investigators have studied *MEFV* mutations in patients with HSP. The aim of the study is to investigate the frequency and clinical significance of MEFV mutations in Egyptian children with Henoch-Schonlein purpura (HSP). Investigating *MEFV* mutations in controls may help in estimating the prevalence of *MEFV* mutation carrier rate in Egyptian children.

**Methods:**

The study enrolled 90 individuals, sixty children with Henoch-Schonlein purpura (HSP), together with 30 sex-and age-matched apparently healthy controls. The entire study group was screened for 12 common *MEFV* mutations using a reverse hybridization assay of biotinylated PCR products.

**Results:**

Patients with HSP had a significantly higher frequency of *MEFV* mutations (61.7%), when compared to the apparently healthy control population (36.7%). V726A was the most frequent mutation with an allelic frequency of 10.8%. Ninety- one percent of patients with *MEFV* mutations were heterozygous for one mutation, while 8.1% had a compound heterozygous MEFV gene mutations. The mutation V726A, followed by E148Q, were the leading mutations, present in 16.6% and in 13.3% of controls.

**Conclusions:**

*MEFV* mutations may be related to HSP susceptibility in children. The mutations were not associated with any clinical and laboratory manifestations. Screening for *MEFV* mutations in larger number of HSP children may be beneficial to evaluate any possible relationship between certain types of *MEFV* mutations and HSP, and compare the HSP *MEFV* mutations to the types of *MEFV* mutations associated with FMF.

**Electronic supplementary material:**

The online version of this article (doi:10.1186/1546-0096-12-41) contains supplementary material, which is available to authorized users.

## Background

Henoch-Schonlein purpura (HSP) is an IgA-mediated immune complex small vessel vasculitis and is the most common systemic vasculitis in childhood. It is characterized by non-thrombocytopenic palpable purpura, abdominal pain, gastrointestinal bleeding, arthritis or arthralgia and nephritis [1–3]. The exact etiology of HSP is not known and no specific genetic abnormalities have been described in HSP patients [4]. Familial Mediterranean fever (FMF) is a monogenic auto-inflammatory disease affecting the Mediterranean population and caused by mutations in the MEFV gene. FMF is characterized by recurrent febrile episodes, pleurisy, peritonitis, arthritis and rash and may be complicated by renal amyloidosis, although wide clinical variability may be present [5, 6]. Several studies reported an increase frequency of MEFV mutations among children of vasculitic and rheumatic diseases, like inflammatory bowel disease (IBD), polyarteritis nodosa (PAN), HSP and juvenile idiopathic arthritis (JIA) [7–9]. FMF has been reported in patients with HSP and other systemic vasculitis. The MEFV gene has been suggested to play an important role in the pathogenesis of this association [7, 10]. Also, *MEFV* mutations have been suggested to exaggerate inflammatory response in HSP [8, 11].

The aim of the present study is to evaluate the frequency of *MEFV* mutations in Egyptian children with HSP and their association with the course and laboratory findings of the disease. Also our aim is to help in estimating the prevalence of *MEFV* mutations carrier rate in Egyptian children.

## Methods

The study enrolled a total of 90 children including 60 patients with HSP diagnosed according to established criteria of the European League against Rheumatism (EULAR) and Pediatric Rheumatology European Society (PReS) [12]. All were followed up at the pediatric rheumatology clinic of the Cairo University Specialized Pediatric Hospital during the period from October 2009 to March 2013. Thirty age-and gender-matched apparently healthy controls, with no family history or clinical manifestations suggestive of HSP or FMF, were assigned to the control group. The age of disease onset had to be before 18 years for the patient to be included in the study. Children with previous diagnosis of FMF prior to the onset of HSP were excluded from the study. The study was approved by the Cairo University Clinical Research Ethics Committee, and informed consents were obtained from parents of all participants. Each enrolled patient was subjected to thorough history taking, including demographic data and disease duration, and also a full physical examination with emphasis on signs of skin, joint, gastrointestinal tract (GIT) and renal involvement were performed. Complete blood count (CBC), erythrocyte sedimentation rate (ESR), C-reactive protein (CRP) concentration, anti-streptolysin O titre (ASOT), blood urea nitrogen, creatinine, urine analysis and occult blood in stool tests were determined by the standard laboratory methods at the time of study enrollment.

### MEFV gene mutation analysis

Blood samples for mutation analysis were also obtained at time of study enrollment from either newly diagnosed patients or previously diagnosed patients following up at the clinic. All children were screened for 12 MEFV gene mutations (E148Q in exon 2, P369S in exon 3, F479L in exon 5, M6801 [G/C], M6801 [G/A], 1692del [2076–2078], M694V, M6941, K695R, V726A, A744S, R761H in exon 10) using the FMF Strip Assay, Vienna Lab Diagnostics GmbH, Vienna, Austria [13]. Genomic DNA was extracted from peripheral blood with ethylenediamine tetraacetic acid (EDTA) by standard procedures. The assay is based on light cycler real-time quantitative polymerase chain reaction (RTPCR) and reverse hybridization, suitable for determination of MEFV mRNA expression. It includes PCR amplification by a thermo cycling program of 35 cycles(94°C for 15 seconds, 58°C for 30 seconds and 72°C for 30 seconds) with final extension at 72°C for 3 minutes, followed by the hybridization of the amplification products to a test strip containing both wild and mutant allele-specific oligonucleotide probes immobilized as an array of parallel lines. Bound biotinylated sequences were detected using streptavidin-alkaline phosphatase and enzymatic color reactions. For each polymorphic position, one of three possible staining patterns were obtained {wild type probe only (normal genotype), wild type and mutant probe (heterozygous genotype), or mutant probe only (homozygous mutant genotype)}.

### Statistical analysis

Chi-Square or Fischer’s exact test (when appropriate) and Odds ratio were used to assess the association between qualitative variables. Quantitative variables were compared by t-student or ANOVA test when appropriate. In all tests, p value was considered significant if less than 0.05. All statistical calculations were done using computer programs SPSS (Statistical Package for the Social Science; SPSS inc., Chicago, IL, USA) version 22 for Microsoft Windows.

## Results

The study included 60 Egyptian children with HSP, 31 females and 29 males. Their mean age at disease onset was 8.1 ± 3 years. The parents of 27% of patients were consanguineous, and 8.1% of patients had family history of FMF. The control group included 30 children with a mean age of 7.67 ± 3. The demographic characteristics of the study group were summarized in Table 1. Clinical and laboratory characteristics of the patients were summarized in Tables 2 and 3. All patients had purpura at the time of diagnosis. Arthritis occurred in 44 patients (73.3%), abdominal pain in 48 (80%), while gastrointestinal complication occurred in 14 (23.3%) and hypertension in 4 (6.7%) patients. Thirteen patients (21.7%) showed recurrence of HSP. MEFV mutations were detected in 37 (61.7%) of HSP patients, with an allelic frequency of 34.1%, and in 11 (36.7%) of the controls with an allelic frequency of (18.33%). A statistically significant difference was detected between patients and controls (p = 0.03). V726A mutation was the leading mutation in HSP patients, with a frequency of 21.7%, while V726A, followed by E148Q, were the leading mutations in controls, with a frequency of 16.6%, and 13.3% respectively. The MEFV genotypic and allelic frequencies are summarized in Table 4. Among the 37 HSP patients with mutated MEFV gene, 34 (91.9%) were heterozygous for one mutation, 3 (8.1%) were compound heterozygous and none of our patients had homozygous mutation (Table 2).

**Table 1:** **Characteristics of HSP patients and controls**

Variable	Cases (n = 60)	Control (n = 30)	P-value
Mean age ± SD	8.1 ± 3	7.67 ± 3	0.66
Range	2-15	5-16	
Sex			
Boys	29(48.3%)	12(40%)	0.45
Girls	31(51.7%)	18(60%)	
*Mutated MEFV gene	37(61.7%)	11(36.7%)	0.03
Sex of children with mutated			
MEFV gene			
Boys	16(43.2%)	6(54.5%)	0.51
Girls	21(56.8%)	5(45.5%)	

*Odd ratio for mutation vs having the disease = 2.8 (95% CI 1.1-6.9).

**Table 2:** **Characteristics of HSP patients stratified by the presence of** ***MEFV*** **mutations**

Variable	Mutation(-)	At least one mutation(+)	P-value
	n = 23 (38.3%)	n = 37 (61.7%)	
Mean age at onset (year)	8.26 ± 3..47	7.93 ± 3.2	0.71
Sex (n,%)			0.32
Boys	13(56.5%)	16(43.2%)	
Girls	10(43.5%)	21(56.8%)	
*F.H. of FMF	0(0%)	3(8.1%)	0.279
Arthritis	16(69.6%)	28(75.7%)	0.603
Abdominal pain	18(78.3%)	30(81.1%)	1
GIT complication	5(21.7%)	9(24.3%)	0.818
Hypertension	3(13%)	1(2.7%)	0.153
Recurrence	5(21.7%)	8(21.6%)	1

*F.H of FMF: family history of Familial Mediterranean Fever.

**Table 3:** **Laboratory investigations of HSP patients stratified by the presence of** ***MEFV*** **mutations**

Variable	Mutation (-)	At least one mutation(+)	P-value
	n = 23 (38.3%)	n = 37 (61.7%)	
*Anaemia	7(30.4%)	9(24.3%)	0.6
*Leukocytosis	10(43.5%)	11(29.7%)	0.278
*Thrombocytopenia	2(8.7%)	1(2.7%)	0.552
ESR(mean ± SD)	36.70 ± 20.007	37.04 ± 27.466	0.96
Positive CRP	11(47.8%)	11(29.7%)	0.157
•Elevated ASOT	7(30.4%)	5(13.5%)	0183
Heamaturia	5(21.7%)	6(16.2%)	0.734
Protienuria	8(34.8%)	7(18.9%)	0..168
Elevated urea and creatinine	0	1(2.7%)	1
Positive stool for occult blood	5(21.7%)	10(27%)	0.646

*Anaemia: HB < 10, leucoytosis: TLC > 11,000cells/cmm, thrombocytopenia: platelet count < 100,000cells/cmm •elevated ASOT > 400.

**Table 4:** ***MEFV*** **gene mutations in HSP patients and controls**

	HSP patients	Controls
	(n = 60)	(n = 30)
Wild-type, [mutation (–)]	23(38.33%)	19(63.3%)
Presence of MEFV gene mutations	37(61.7%)	11(36.7%)
Heterozygous for one		
mutation		
p.V726A / –	12(20%)	5(16.6%)
p.E148Q / –	8(13.3%)	4(13.3%)
p.M680I (G/A) / –	8(13.3%)	1(3.33%)
p.M694V / –	5(8.3%)	1(3.33%)
P369S / –	1(1.7%)	
Compound heterozygous for two or three mutations:	3(5%)	
E148Q/M694V	2(3.3%)	
M680I/M694V/V726A	1(1.70%)	
Allelic Frequency of MEFV gene mutations		
	**HSP patients**	**Controls**
	**alleles(n = 120)**	**alleles(n = 60)**
V726A	13(10.83%)	5(3.15%)
E148Q	10(8.3%)	4(2.5%)
M680I (G/A)	9(7.5%)	1(.63%)
M694V	8(6.6%)	1(.63%)
P369S	1(0.83%)	
Total	41(34.1%)	11(18.33%)

When stratified according to the presence or absence of MEFV gene mutations, no statistically significant differences were detected between the 2 groups; regarding demographic, clinical and laboratory characteristics (Tables 2 and 3). Although arthritis, abdominal pain, GIT complication and stool positivity for occult blood were all more frequently observed in patients with MEFV gene mutations, differences were not statistically significant. Eight of 37 patients (21.6%) with *MEFV* mutation and five of 23 patients (21.7%) without mutations had one or more recurrence of their disease with no significant difference (Table 3). When stratified according to the presence of V726A gene mutation , non-V726A gene mutation and wild type mutation, no association was detected between the type of mutation and demographic, clinical and laboratory characteristics (Additional file 1: Table S1 and Additional file 2: Table S2).

## Discussion

Due to an increased frequency of vasculitis in FMF patients, many investigators have studied *MEFV* mutations in patients with HSP [8, 9, 11, 14]. In the present study, *MEFV* mutations were detected in 61.7% of HSP patients, with a statistically significant difference between patients and controls (p = 0.03). This high frequency of *MEFV* mutations among HSP patients is consistent with the results of other studies [8, 11, 14, 15].

The frequency of MEFV gene mutation in our HSP study group is higher than the carrier rate of *MEFV* mutation among Egyptian general healthy population (18.4%), reported in a limited number of studies [16]. The frequency of MEFV gene mutations in our HSP study group was more than its frequency in Turkey, as reported by Bayram et al. (44% [11]),and Ozcakar et al. (34% [14]). Also Dogan et al. detected MEFV gene mutations in 24.3% of HSP children, with an allelic frequency of 16.8% [15]. Our results were also higher than the results of Gershoni-Baruch et al. who examined 52 HSP children in Israel (30 Arabs, 22 Jews). These MEFV gene mutations were detected in 27%, with an allelic frequency of 18.2% [8]. The frequency of MEFV mutations in our children with HSP is surprisingly high. This difference may be related to ethnic differences, variations in number of recruited patients in each study, a higher rate of consanguineous marriage, and variations in study design. The frequency of MEFV gene mutation in Egyptian patients with HSP in the present work (61.7%) is very close to its frequency in Egyptian children with FMF, reported as (57.6%) in the study of El Gezery et al. [17] and 60.5% in the work of Ibrahim et al. [18], but lower than the frequency in the work of El Garf et al. (97%) [19]. These results may point to the presence of association between HSP and FMF, but determining such association will require a larger sample size of HSP and FMF patients, optimally including a subgroup of HSP patients with a previous history of FMF for comparison.

In the present study, mutations detected in 34 (91.8%) of HSP patients were heterozygous ,while only 3(8.1%) had compound heterozygous MEFV mutations, which is consistant with the results of Bayram et al. [11] in which (70%) of HSP patients with *MEFV* mutations had heterozygous mutations, 12.7% had compound heterozygous mutations and 17% had homozygous mutations. Heterozygous mutations were also more prevalent among HSP patients with MEFV gene mutations in the study of Gershoni-Baruch et al. [8]. V726A was the commonest mutation detected in our HSP study group, which is not consistent with the results of other Turkish studies where M694V was the most common mutation [11, 14]. Our results are also not consistant with the results of Gershoni et al. [8] from Israel and He et al. [20] from China in which E148Q being the most frequent mutation among patients with HSP (43% and 85%) respectively. In a study of Iranian HSP patients, V726A mutation was detected in only 2% of HSP patients with detected *MEFV* mutations, while the M694V mutation was the most frequent mutation (22%) [21]. The variation in the type of genetic mutation between our HSP patients and patients from other countries may be related to the genetic heterogeneity of the Egyptian population. This population is one that has undergone genetic admixture and racial mixing, which created a heavily mixed population of modern Egyptians including several ethnic groups such as Bedouins, Peasants, Nubians, Berbers, and urbanites [22]. This mutational heterogeneity appears to be less obvious among other ethnic populations. The comparison of mutations in HSP patients with history of FMF may ultimately be beneficial in determining the type of mutation predisposing to FMF and the types of *MEFV* mutations protecting against FMF.

The V726A genetic mutation is the most common mutation in our HSP study group, yet among Egyptian FMF patients, the most common genetic mutations are quite different including M6941 in 2 studies [18, 23], E148Q in another study [24] ,and M694V in a recent work of Al- Haggar et al. in 2014 [25]. While our work does suggest that the most frequent *MEFV* mutations associated with HSP is different from the most common mutations associated with FMF, a study of a much larger number of HSP patients is needed to confirm this finding.

When stratified according to the presence of MEFV gene mutations, no statistically significant differences in clinical manifestations and laboratory findings were detected between HSP patients, similar to the result reported by Gershoni et al. [8]. In contrast, Bayram et al. [11] and Ozcakar et al. [14] reported that the presence of *MEFV* mutations may affect the clinical manifestations and laboratory findings in HSP.

Our results show that 36.7% of our studied healthy controls were carriers for *MEFV* mutations with an allelic frequency of 18 .33%. V726A, followed by E148Q, were most common mutations. These results are higher than the results of Al-Alami et al. [16], who reported an allelic frequency of MEFV gene mutation among apparently healthy mixed Arabic population to be 9.3%. It is also higher than the carrier rate in the Syrian population (17.5%), with E148Q being the leading mutation, followed by V726A and M694V [26]. Our MEFV carrier rates are also higher than the carrier rate found in an Algerian study (19.13%), with E148Q being the commonest MEFV gene mutation [27].

The heterogeneity in the genetic mutations between Egyptian and other Mediterranean countries may be due to the strategic position of Egypt as a crossroad between countries, causing a real genetic admixture within the Egyptian population [28]. Unfortunately, the small number of studied individuals in the available studies have not been statistically capable of definitively determining the carrier rate of *MEFV* mutations in Egypt. To our knowledge, this is the first study examining the frequency of *MEFV* mutations in HSP children in Egypt. Egypt remains an understudied population for these mutations. As noted, one of the main limitations of the study is that the present study doesn’t have the statistical power to determine the relationship of certain *MEFV* mutations with HSP susceptibility or disease phenotype or outcomes. Larger number of studied HSP patients and controls may be needed in future studies. Further studies with larger numbers of apparently healthy children may be also required to establish a data base for the carrier rate of *MEFV* mutations in Egypt.

## Conclusion

Our study suggests that the *MEFV* mutations are much more frequent in Egyptian HSP children than healthy controls, especially the V726A mutation. The presence of *MEFV* mutations in our HSP children is not associated with a statistically significant difference in clinical presentation and laboratory findings. When correlating our results to other studies from the literature, we believe that the next step is to study this possible association between HSP and FMF MEFV gene mutations in a much larger number of Egyptian HSP children.

## Electronic supplementary material

Additional file 1: Table S1: Characteristics of HSP patients with *MEFV* mutations stratified according to type of mutation. (DOCX 18 KB)

Additional file 2: Table S2: Laboratory investigations of HSP patients with *MEFV* mutations stratified according to type of mutation. (DOCX 18 KB)
