# Supplementary material for: MEFV gene mutations in Egyptian children with Henoch-Schonlein purpura
Source: Pediatr Rheumatol Online J. 2014 Sep 9;12:41. doi: 10.1186/1546-0096-12-41 (PMC4165914; doi:10.1186/1546-0096-12-41)
Supplement: Supplementary file 1 — Additional file 1: Table S1: Characteristics of HSP patients with MEFV mutations stratified according to type of mutation. (DOCX 18 KB) [file 12969_2014_1741_MOESM1_ESM.docx]

**Additional file 1: Table S1.** Characteristics of HSP patients with MEFV mutations stratified according to type of mutation

|  | V726A mutation | Non V726A mutation | Without mutation | P-value |
| --- | --- | --- | --- | --- |
|  | n = 13 | n = 24 | n = 23 |  |
|  | (21.7%) | (40%) | (38.3%) |  |
| Mean age at onset | 9.61 ± 3.39 | 7. 02 ± 2.74 | 8.26 ± 3.46 | 0. 064 |
| Sex |  |  |  | 0.325 |
| Boys | 4 (30.8%) | 12 (50%) | 13 (56.5%) |  |
| Girls | 9 (69.2%) | 12 (50%) | 10 (43.5%) |  |
| F.H of FMF | 2 (15.4%) | 1 (4.2%) | 0 | 0.123 |
| Hypertension | 0 | 1 (4.2%) | 3 (13%) | 0.263 |
| Arthritis | 8(61.5%) | 20(83.3%) | 16 (69.6%) | 0.314 |
| Abdominal pain | 12 (92.3%) | 18 (75%) | 18 (78.3%) | 0.438 |
| GIT complication | 3 (23.1%) | 6 (25%) | 5 (21.7%) | 0.965 |
| Recurrence | 4 (30.8%) | 4 (16.7%) | 5 (21.7%) | 0.610 |
