# Supplementary material for: MEFV gene mutations in Egyptian children with Henoch-Schonlein purpura
Source: Pediatr Rheumatol Online J. 2014 Sep 9;12:41. doi: 10.1186/1546-0096-12-41 (PMC4165914; doi:10.1186/1546-0096-12-41)
Supplement: Supplementary file 2 — Additional file 2: Table S2: Laboratory investigations of HSP patients with MEFV mutations stratified according to type of mutation. (DOCX 18 KB) [file 12969_2014_1741_MOESM2_ESM.docx]

**Additional file 2: Table S2.** Laboratory investigations of HSP patients with MEFV mutations stratified according to type of mutation

|  | V726A mutation | Non V726A mutation | Without mutation | P-value |
| --- | --- | --- | --- | --- |
|  | n = 13 | n = 24 | n = 23 |  |
|  | (21.7%) | (40%) | (38.3%) |  |
| Anaemia | 3 (23.1%) | 6 (25%) | 7 (30.4%) | 0.866 |
| Leucocytosis | 4 (30.8%) | 7 (29.2%) | 10 (43.5%) | 0.552 |
| Thrombocytopenia | 1 (7.7%) | 0 | 2 (8.7%) | 0.346 |
| ESR (mean ± SD) | 33.38 ± 19.9 | 38.5 ± 20.2 | 37.04 ± 27.4 | 0.815 |
| Positive CRP | 4 (30.8%) | 7 (29.2%) | 11 (47.8%) | 0.366 |
| Elevated ASOT | 1 (7.7%) | 4 (16.7%) | 7 (30.4%) | 0.227 |
| Hematuria | 3 (23.1%) | 3 (12.5%) | 5 (21.7%) | 0.632 |
| Proteinuria | 3 (23.1%) | 4 (16.7%) | 8 (34.8%) | 0.352 |
| Elevated urea and creatinine | 0 | 1 (4.2%) | 0 | 0.466 |
| Positive stool for occult blood | 2 (15.4%) | 8 (33.3%) | 5 (21.7%) | 0.436 |
